# Methyl-qPCR: a new method to investigate Epstein–Barr virus infection in post-transplant lymphoproliferative diseases

**DOI:** 10.1186/s13148-022-01255-1

**Published:** 2022-03-04

**Authors:** Chloé Borde, Frédérique Quignon, Corinne Amiel, Joël Gozlan, Vincent Marechal, Eolia Brissot

**Affiliations:** 1 Sorbonne Université, INSERM U938, Centre de Recherche Saint-Antoine, 75012 Paris, France; 2 Sorbonne Université, CNRS UMR 144, Institut Curie, Paris, France; 3 AP-HP, Service de Virologie, Hôpital Tenon, 75020 Paris, France; 4 Sorbonne Université, Service de Virologie, AP-HP, Hôpital Saint-Antoine, 75012 Paris, France; 5 Sorbonne Université, INSERM, Centre de Recherche Saint-Antoine (CRSA), 75012 Paris, France; 6 AP-HP, Hôpital Saint-Antoine, Service d’Hématologie Clinique Et Thérapie Cellulaire, 75012 Paris, France

**Keywords:** Epstein–Barr virus, DNA methylation, Epigenetics, Post-transplant lymphoproliferative disease

## Abstract

Epstein–Barr virus DNA viral load is used as a surrogate marker to start Rituximab in transplant recipients at risk of developing PTLD. However, an elevated EBV DNAemia does not discriminate lymphoproliferation and replication. We designed a new molecular assay (methyl-qPCR) to distinguish methylated versus unmethylated viral genomes. In blood, viral genomes were highly methylated in EBV primary infections, PTLD and 4/5 transplant recipients with high viral load. The only patient with under-methylated EBV genomes did not respond to rituximab. Methyl-qPCR is a convenient method to discriminate between latent and lytic EBV genomes and could be useful in treatment decisions.

**Supplementary Information:**

The online version contains supplementary material available at 10.1186/s13148-022-01255-1.

## Background

Transplant recipients are at high risk of developing Epstein–Barr virus (EBV)-associated Post-Transplant Lymphoproliferative Diseases (PTLD), a group of lymphoid or plasmacytic proliferations that develops as a consequence of immunosuppression after solid organ or hematopoietic stem cell transplantation (HSCT) [1–3]. EBV life cycle alternates latent and lytic phases. Latency has been associated with the expression of a limited set of genes required for viral persistency and cell transformation, whereas the lytic phase is required for the production of viral particles. EBV DNA viral load (EBV VL) in whole blood (WB) or plasma is widely used in high-risk HSCT patients as a surrogate marker to identify sub-clinical PTLD and to start a pre-emptive treatment with the anti-CD20 rituximab [4–6]. However, there is no clear consensus regarding the threshold of EBV DNA that should lead to the start of pre-emptive therapy. In addition, EBV VL, albeit sensitive, keeps a poor positive predictive value for the occurrence of PTLD, which leads to an excess of rituximab therapy [7, 8]. In a non-mutually exclusive manner, an elevated EBV VL may result from an increase in the number of circulating EBV-positive lymphocytes (EBV latent phase), and/or an increase in EBV lytic replication (EBV reactivation), which raises important questions regarding the origin of increased viral load: lymphoproliferation and/or EBV replication [8].

It has been demonstrated that the viral genome is heavily methylated on specific sites in EBV-infected cancer cells and latently infected cell lines [9, 10]. Conversely, EBV genomes are under-methylated in cells undergoing lytic replication as well as in viral particles [10]. Using a combination of methyl-sensitive restriction enzymes and PCR on specific regions of the viral genome, we designed a new and convenient molecular assay thereafter named methyl-qPCR that allows the differential quantification of methylated vs unmethylated viral genomes as a reflect of the relative proportion of latent versus lytic viral genomes in clinical samples.

## Methods

### Cells and clinical samples

The EBV-positive cell line Akata (in a latent cycle) was cultured in RPMI 1640 medium (Gibco BRL) supplemented with 10% v/v heat-inactivated fetal calf serum and 2 mM L-glutamine (Life Technologies). Reactivation in Akata cells to induce a replicative cycle was induced by 7.5 µg/mL polyclonal rabbit anti-human IgG (A0423, Dako), in 0.5% fetal calf serum supplemented medium. Intracellular DNA was collected after 24 h of treatment and directly quantified by a methyl-sensitive qPCR.

The clinical samples were as follows: EBV-positive saliva from healthy subjects (*n* = 10), and whole blood from patients with EBV primary infection (*n* = 9), from patients with confirmed PTLD post HSCT (*n* = 8) and from patients with high EBV VL (> 5000 UI/mL) post-HSCT but no proof of PTLD (*n* = 5).

### Genomic DNA extraction

Total genomic DNA was extracted and purified using DNeasy Blood and Tissue Kit (QIAGEN) for EBV-positive cell lines and saliva, and Blood and cell culture DNA Mini Kit (QIAGEN) for whole blood and plasma, according to the manufacturer’s instructions.

### EBV regions of interest

The relative methylated/unmethylated status of specific sites on viral DNA can be used to quantify the relative proportion of latent vs nonreplicated/virion lytic DNA. A set of four regions (designed as BZLF1, BALF5, LF2 and BDLF2) comprising CCGG sequences were identified from previous work by Fernandez and colleagues [10] that were methylated on latent genomes and unmethylated in lytically replicated DNA. MspI and HpaII are isoschizomers with differing sensitivities to CpG methylation. MspI cleaves both unmethylated and methylated CCGG sites, whereas HpaII cleaves only the unmethylated form of the restriction site. Specific primers were designed around each region of interest to amplify DNA following incubation with each enzyme (see Additional file 1: Figure S1).

### Methyl-sensitive qPCR

Total DNA was subjected to digestion, at 37 °C for 16 h in 1X Cut Smart buffer (Biolabs) with either 100 units of HpaII (R0171M, Biolabs) or 100 units of MspI (R0106M, Biolabs) [11], therefore providing a positive control (Additional file 1: Figure S1A). Then, the four regions of interest were quantified by real-time PCR using TaqPath QPCR (ThermoFisher) with specific primers (Additional file 1: Figures S1B, S2). Cellular ß-globin was co-amplified as a positive control and to allow normalization of EBV DNA quantitation. Reactions were performed on a Biorad CFX96 Touch Real-Time PCR machine. The methylation index was defined according to the 2^−∆∆Ct^ method proposed by Livak and Schmittgen [12]. The methylation index is equal to 2^∆∆CT^, where ∆∆CT is equal to ∆CT_untreated_ (cycle threshold [CT] of the EBV target gene minus CT of the ß-globin reference gene measured on the untreated sample) minus ∆CT_treated_ (CT of the EBV target gene minus CT of the ß-globin reference gene measured on HpaII treated sample). Therefore, the methylation index is equal to 2^(∆CTuntreated−∆CTtreated)^. When the target is methylated, HpaII treatment has no effect and the methylation index is close to 1. When the target is demethylated, i.e., accessible to degradation by HpaII, 2^∆∆CT^ tends to 0. For reasons related to experimental variations in CT measurement, the methylation index may occasionally be higher than 1 for highly methylated targets. Since MspI is degrading both methylated and unmethylated target, 2^∆∆CT^ should always be close to 0 for MspI treated samples, providing evidence that the HpaII/MspI restriction site is present in the target EBV sequence (Additional file 1: Figure S1A).

## Results

### Methyl-qPCR can distinguish between latent and lytic viral DNA in EBV-infected cell lines

**Fig. 1:**
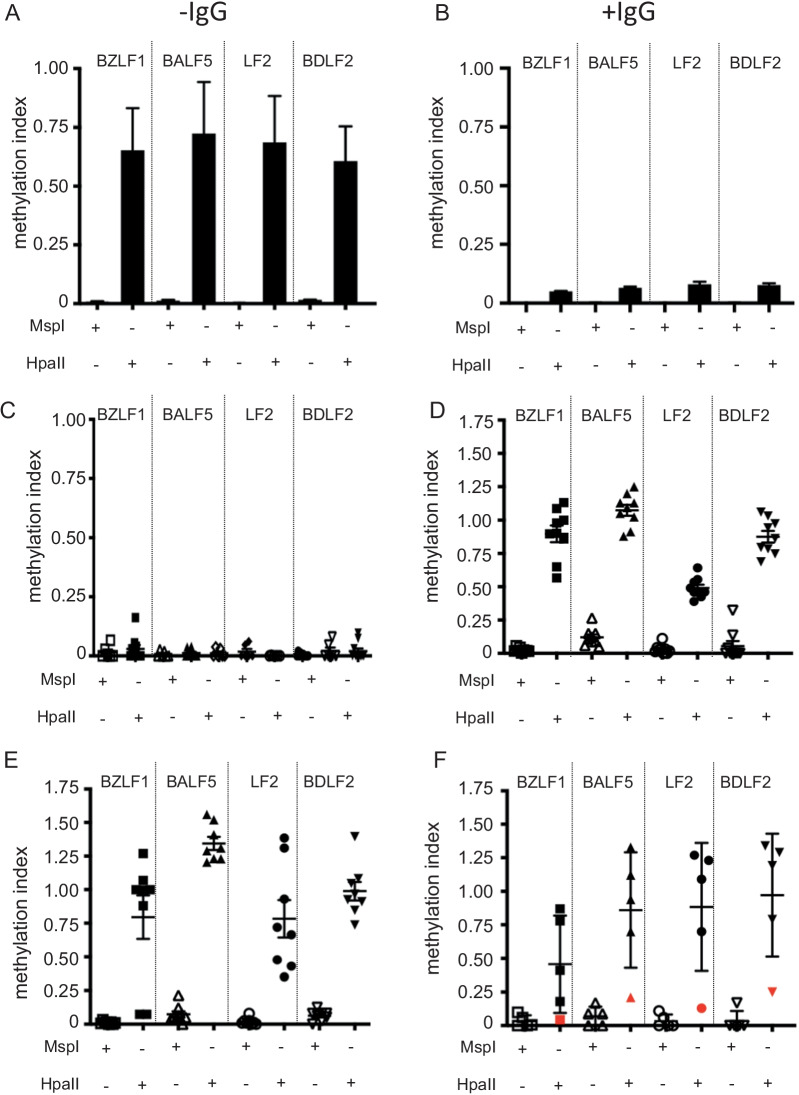
Evaluation of latent versus lytic EBV DNA by methyl-qPCR. Methylation Index was measured on various EBV containing samples. EBV + Akata cell line during latency (**A**) and reactivation (**B**). EBV-positive saliva from healthy patients (*n* = 10) (**C**). Peripheral blood from patients with primary EBV infection (*n* = 9) (**D**); Peripheral blood from confirmed PTLD (post-transplant lymphoproliferative diseases) (*n* = 8) (**E**); Peripheral blood from HSCT recipients with no proof of PTLD (*n* = 5) (**F**). All samples were submitted to methyl-sensitive qPCR on four distinct regions of the viral genome. Each dot represents one individual sample. Whole extracted DNA were digested with either MspI or HpaII, two isoschizomers with different sensitivities to CpG methylation. HpaII is methylation sensitive, whereas MspI is methylation insensitive. For each region specified above, results are expressed in comparison with non-digested DNA. Data are expressed as mean ± SD

The relative level of methylation within 4 EBV regions was first evaluated on EBV-infected cell line Akata both during latency and following lytic reactivation by anti-surface IgG. As shown on Fig. 1A, B, the methylation index ranged between 0.6 and 1 for each region in non-reactivated cells and decreased between 0.04 and 0.09 in reactivated Akata cells. These results show that methyl-qPCR is able to discriminate latent from replicative virus in EBV cell lines).

### Methyl-qPCR reveals a replicative EBV pattern in saliva

The presence of infectious viral particles in saliva is well established. To confirm that methyl-qPCR could efficiently discriminate methylated and unmethylated EBV DNA in vivo, saliva from 10 EBV-positive donors with detectable VL were subjected to methyl-qPCR. EBV DNA from all samples was unmethylated in the four tested regions, independently of the viral load (Fig. 1C; Additional file 1: Table S3). This result confirms that methyl-qPCR could identify unmethylated viral from EBV particles in saliva.

### Methyl-qPCR during EBV primary infection and in HSCT patients with high VL

We tested blood samples from 9 patients with EBV primary infection. As illustrated on Fig. 1D (and Additional file 1: Table S4A), most of the EBV regions tested were highly methylated (mean methylation index ranging from 0.69 to 0.93) indicating that mostly latent genomes account for the detection of EBV VL in peripheral blood during EBV primary infection.

Whole blood analysis was also performed in 13 samples from HSCT patients with high VL (8 with confirmed PTLD and 5 with no proof of PTLD but high VL). For PTLD patients, the high methylation index in most EBV regions tested confirmed that EBV DNA was under a latent form indicating that the high viral load was indeed due to the proliferation of cells containing latent genomes in these patients (Fig. 1E and Additional file 1: Table S4B). A high methylation index was also measured in 4 out of 5 patients with high EBV VL but no proven PTLD (Fig. 1F and Additional file 1: Table S4C). Only one patient displayed a low methylation index in the four regions tested. Interestingly, this patient kept a high EBV VL despite eight injections of rituximab suggesting that a high viral load originating from lytically infected cells might be less sensitive to B cell destruction mediated by anti-CD20 antibody.

Importantly, no correlation was observed between the level of methylation and the viral load, whatever the viral region or the patients (data not shown).

## Discussion

**Fig. 2:**
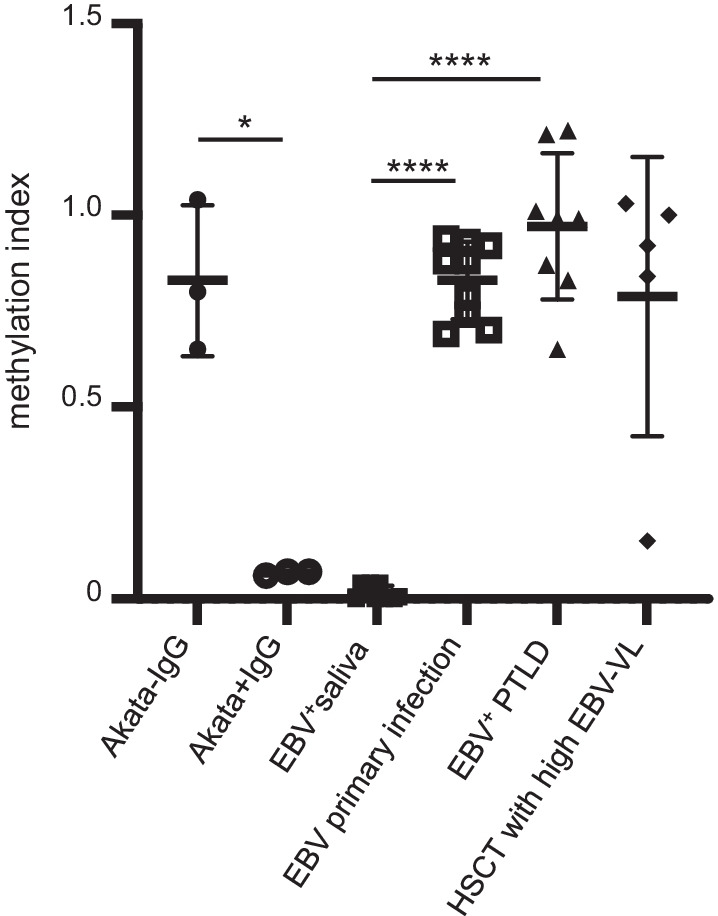
Representation of mean methylation index in Akata cells during latency and reactivation; saliva (*n* = 10) or blood of patients with primary infection (*n* = 9); confirmed PTLD (post-transplant lymphoproliferative diseases) (*n* = 8); or no proven PTLD (*n* = 5). Each dot represents one individual sample. Data are expressed as mean ± SD. *p < 0.05; ****p < 0.0001

Altogether, we showed herein that methyl-qPCR is a convenient method to discriminate between latent and lytic EBV genomes in biological samples (Fig. 2). This proof-of-concept study, which was applied to a small number of patients, confirmed that EBV load in samples from IM, from PTLD or from suspected PTLD samples are mostly methylated, i.e., issued from latently infected cells. A larger cohort analysis on allogenic HSCT recipients with high EBV VL is now undertaken to confirm these preliminary data, to compare methylation status of EBV DNA in WB and plasma and to analyze the correlation between DNA methylation and response to a pre-emptive therapy with rituximab. We believe that this method will allow a more accurate selection of patients with high EBV VL who should benefit from rituximab therapy.

## Supplementary Information

**Additional file 1**: Figure S1.

## Abbreviations

EBV: Epstein–Barr virus
HSCT: Allogeneic stem cell transplantation
PTLD: Post-transplant lymphoproliferative disease
VL: Viral load
