# Supplementary material for: Methyl-qPCR: a new method to investigate Epstein–Barr virus infection in post-transplant lymphoproliferative diseases
Source: Clin Epigenetics. 2022 Mar 4;14:33. doi: 10.1186/s13148-022-01255-1 (PMC8895795; doi:10.1186/s13148-022-01255-1)
Supplement: Supplementary file 1 — Additional file 1: Figure S1. [file 13148_2022_1255_MOESM1_ESM.pdf]

Supplemental data

A.

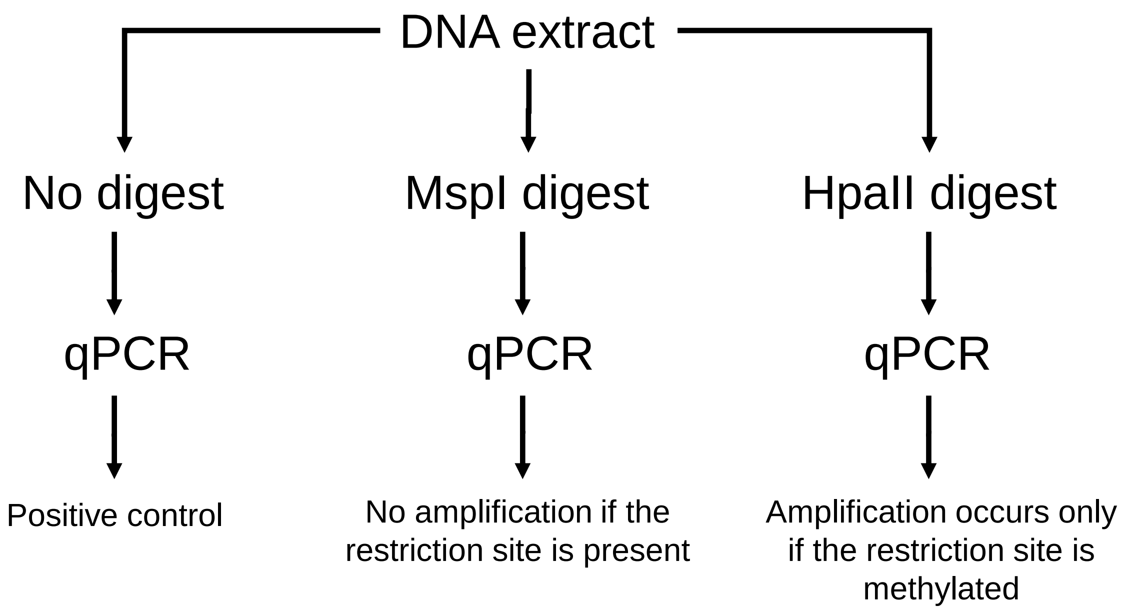

B.

region	Forward primer	Reverse primer	probe for Taq Man
BZLF1	CTGCTGGTTGAACAGTAGAATTG	GTTCCCAGTCTCAGACATAACC	CCCGGCTTGGTTAGTCTGTTGATTCT
BALF5	GGATCGTGATAGCGTCTTCTG	TAGCGCTGCATGAGCAAA	CGCCCTCTGAGCTGCTCTCTTT
LF2	ACGCTAGTGCTGCATGG	TAACGAGCGGAGAGTTGTATTG	TCGACGGGCAAGGTGAGAGT
BDLF2	GATTCAAGAGGTCGTGATGCTC	CTTGGCCCTGCTGGACA	CAGCAGGGTCACCATGATGCAA
Bglobin	TAGCAACCTCAAACAGACACCA	TCACCACCAACTTCATCCAC	CAGGGCAGTAACGGCAGACTTCTC

Figure S1
The relative quantification of methylated/undermethylated DNA on specific sites can be used to distinguish between latent and nonreplicated/virion lytic DNA **(A).** We selected a set of 4 regions (thereafter designed as BZLF1, BALF5, LF2 and BDLF2) comprising CCGG sequences that were methylated on latent genomes and unmethylated in lytic viral DNA. MspI cleaves both unmethylated and methylated CCGG site whereas HpaII is inhibited by methylation. Specific primers were designed around each region of interest to amplify residual DNA following incubation with each enzyme **(B).**

primers	slope	PCR efficiency
BZLF1	3.536	1.917
BALF5	3.15	2.077
LF2	3.351	1.988
BDLF2	3.33	1.996

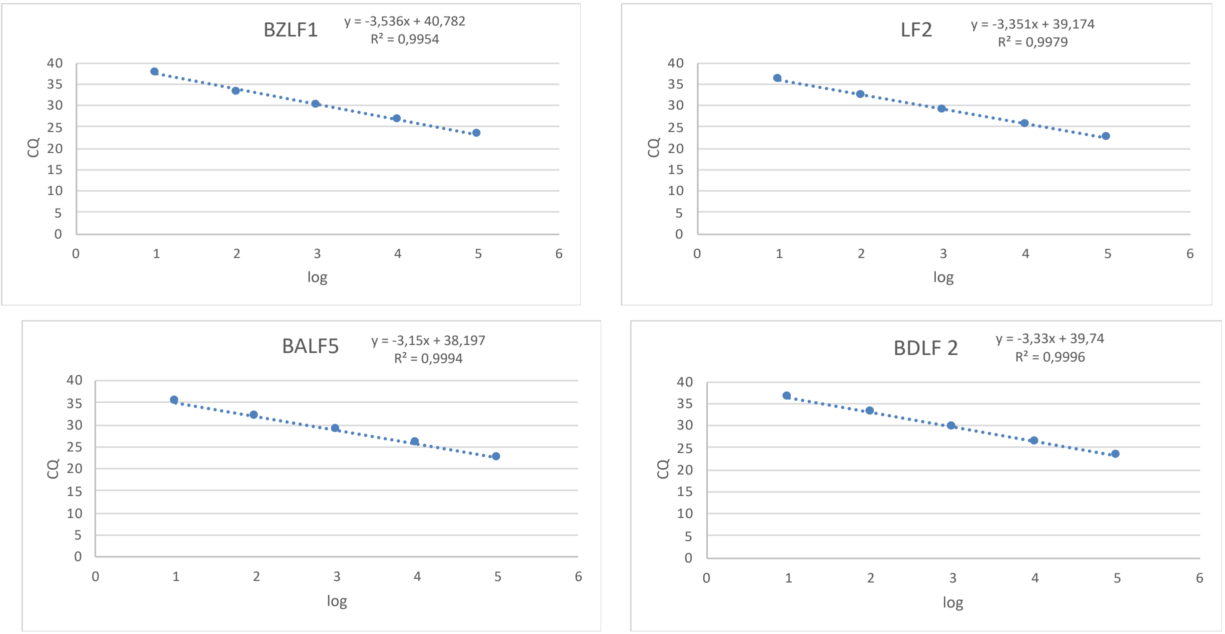

Figure S2
PCR efficiency and slope values for the four EBV regions (BZLF1, LF2, BALF5 and BDLF2) targeted by methyl-qPCR

Patient (saliva)	Viral load (c/ml)	BZLF1	BALF5	LF2	BDLF2	Methylation index
1	55 913	0.04	0.02	0.01	0.09	0.04
2	1 336	0	0	0	0	0
3	3 241	0.16	0	0	0	0.04
4	1 738	0	0	0	0	0
5	6 585 126	0.05	0.04	0.01	0.07	0.04
6	2 332	0	0	0	0	0
7	23 293	0	0.02	0	0	0.005
8	92 935	0.01	0	0	0	0.0025
9	3 326	0	0	0	0	0
10	2 735 626	0.03	0.04	0.02	0.03	0.03
Median	13 310	0.005	0	0	0	0.003
Mean	950 487	0.03	0.02	0.003	0.02	0.015

Figure S3

The characteristics of EBV+ patients (saliva) are summarized: viral load (EBV-R-GENE^{R}-Biomerieux), methylation index for each region and the average methylation index for each sample

A.

Patient (MI)	Viral load (c/ml)	BZLF1	BALF5	LF2	BDLF2	Methylation index
1	164 567	0.86	1.25	0.46	0.97	0.88
2	60 091	0.65	0.88	0.39	0.86	0.69
3	219 565	1.08	1.20	0.55	0.94	0.94
4	19 182	0.90	1.02	0.46	0.79	0.79
5	38 039	1.13	1.05	0.53	1.03	0.93
6	14 437	0.97	1.12	0.64	0.78	0.88
7	41 305	0.56	1.09	0.45	0.68	0.70
8	124 371	0.89	0.91	0.42	0.74	0.74
9	15 187	1.00	1.12	0.49	1.06	0.92
median	41 305	0.90	1.09	0.46	0.86	0.88
mean	77 416	0.89	1.07	0.49	0.87	0.83

B.

Patient (PTLD)	Viral load (c/ml)	BZLF1	BALF5	LF2	BDLF2	Methylation index
1	2 047 148	1.00	1.56	0.66	0.84	1.01
2	17 701	0.88	1.23	0.47	0.91	0.87
3	6 036	1.00	1.31	0.72	0.96	0.99
4	9 642	1.07	1.52	0.35	1.04	0.99
5	6 036	1.27	1.29	0.93	1.39	1.22
6	8 173 909	0.07	1.20	0.43	0.92	0.65
7	1 391 743	0.99	1.40	1.38	1.08	1.21
8	412 984	0.07	1.23	1.31	0..73	0.83
Median	215 343	1.00	1.29	0.66	0.96	0.99
Mean	1 508 150	0,81	1.32	0.75	0.99	0.97

C.

Patient (HSCTwith high EBV VL)	Viral load (c/ml)	BZLF1	BALF5	LF2	BDLF2	Methylation index
1	25 952	0.78	0.70	1.09	0.79	0.84
2	4 932	0.18	1.33	1.23	1.29	1.00
3	32 915	0.41	1.12	1.27	1.34	1.03
4	48 575	0.04	0.21	0.13	0.25	0.15
5	91 230	0.87	0.94	0.70	1.19	0.92
Median	32 915	0.41	0.94	1.09	1.19	0.92
Mean	40 721	0.45	0.86	0.88	0.97	0.78

Figure S4
The characteristics of EBV+ patients with EBV primary infection **(A);** PTLD (post-transplant lymphoproliferative diseases) **(B)**; or no proof of PTLD **(C)** are summarized: viral load; methylation index for each region and the average methylation index for each sample.
